# Discussing on the Aortic Coverage in Type B Aortic Dissection Treatment: A Comprehensive Scoping Review

**DOI:** 10.3390/jcm13133897

**Published:** 2024-07-02

**Authors:** Daniele Bissacco, Jasper F. de Kort, Anna Ramella, Sara Allievi, Paolo Bellotti, Renato Casana, Maurizio Domanin, Francesco Migliavacca, Santi Trimarchi

**Affiliations:** 1 Department of Clinical Sciences and Community Health, University of Milan, 20148 Milan, Italy; maurizio.domanin@unimi.it (M.D.); santi.trimarchi@unimi.it (S.T.); 2 Section of Vascular Surgery, Cardio Thoracic Vascular Department, Foundation IRCCS Ca’ Granda Ospedale Maggiore Policlinico, 20122 Milan, Italy; jasperdkort@gmail.com (J.F.d.K.); paolo.bellotti@unimi.it (P.B.); 3 Department of Chemistry, Materials and Chemical Engineering “G. Natta”, Politecnico di Milano, 20133 Milan, Italy; anna.ramella@polimi.it (A.R.); francesco.migliavacca@polimi.it (F.M.); 4 Department of Vascular Surgery, Santa Chiara Hospital, 38122 Trento, Italy; sara.allievi3@gmail.com; 5 Department of Cardiology, Istituto Auxologico Italiano, IRCCS, 20145 Milan, Italy; r.casana@auxologico.it

**Keywords:** scoping review, thoracic aorta, aortic dissection, TEVAR

## Abstract

Objective: The objective of this study is to investigate and address the question surrounding the determination of the optimal endograft length of coverage during TEVAR for type B aortic dissection (TBAD), with a particular emphasis on the distal landing zone (DLZ). Data sources: MEDLINE, Scopus, and Web of Science databases were used. Methods: The PRISMA-ScR statement was followed. Results: Several variables can contribute to the length of coverage during TEVAR in TBAD patient. Baseline patient’s characteristics, TBAD-related features, the type of endoprosthesis, and postoperative graft behaviour may contribute to the choice of coverage. Conclusions: No robust data have been published regarding the optimal length of TEVAR. Therefore, reporting the percentage of covered aorta and improving computational studies should be valorised to improve postoperative outcomes.

## 1. Introduction

Thoracic endovascular aortic repair (TEVAR) has been firmly established as the preferred treatment option for both descending thoracic aorta aneurysm (DTAA) and type B aortic dissection (TBAD). One of the keys for obtaining the success of the endovascular procedure and the reduction of postoperative complications relies on meticulous preoperative planning. This planning process involves the careful consideration of various factors, including the anatomical and morphological features of the aorta as well as the type and extent of the disease.

In the case of TBAD, the evaluation of landing zones (LZs) during preoperative planning is of the utmost importance to ensure positive perioperative and long-term outcomes. However, the criteria for treatment success differ between aortic aneurysms and dissections. For aortic aneurysms, the ideal landing zone is considered to be a healthy segment of the aorta, and the success of TEVAR is measured by the elimination of blood flow in the aneurysm sac, avoiding its pressurization after TEVAR. In contrast, for patients with aortic dissection, TEVAR success is defined by the closure of the entry tear/tears, the promotion of blood flow in the true lumen (TL), and the induction of thrombosis in the false lumen (FL).

Furthermore, for both DTAA and TBAD, considerable attention should be given to postoperative short- and long-term aortic remodelling, which occurs as a result of the hemodynamic changes caused by the TEVAR procedure [1]. Remodelling effects arise from modifications in the shape of the native aorta following the procedure and/or the interactions between the graft and the native aortic wall. It is important to avoid areas of mechanical and/or hemodynamical conflicts, where the forces exerted by the graft may have detrimental effects on the aorta, which may potentially be causes of complications, limiting the benefits of the endovascular treatment [2]. These measures acquire greater importance and complexity in the case of TBAD, in which the dissected wall expresses different compliance and resistance to the endograft.

In this context, both the proximal landing zone (PLZ) and the distal landing zone (DLZ) must be carefully considered and analysed. While the PLZ ensures a secure and effective sealing of the graft, the DLZ aims to provide sufficient coverage to optimize outcomes and minimize postoperative complications in the distal region.

However, the guidelines do not offer specific recommendations regarding the management of the DLZ, leaving uncertainty in the literature regarding how to define the concept of “covering enough” [3,4,5]. In other words, the optimal length of TEVAR in TBAD, which strikes a balance between the potential benefits and harms, remains inadequately defined. On one hand, it may not be prudent to always cover the entire thoracic or thoracoabdominal aorta to minimize the risk of complications affecting the spinal cord or splanchnic region. On the other hand, it has been reported as safe and useful to cover the total descending aorta in order to promote positive remodelling in this aortic segment.

The objective of this scoping review is to delve into the question of determining the appropriate endograft length of coverage during TEVAR for TBAD, with a specific focus on the DLZ. The review comprehensively analyses patient-related factors, aortic characteristics, technical considerations, and graft-related variables that could influence the determination of the length of coverage and so the position of DLZ, aiming to provide insights and guidance in this important aspect of TEVAR planning for TBAD.

## 2. Materials and Methods

### 2.1. Design

A scoping review was performed according to the Preferred Reporting Items for a Systematic Review and Meta-Analysis Protocols (PRISMA-P) Extension for Scoping Reviews (PRISMA-ScR) [6]. The complete checklist can be found in Appendix A. The decision to undertake a scoping review was prompted by the observation that upon initial examination of the available literature, the findings appeared to be highly diverse and lacking consistency. Conducting a scoping analysis seemed necessary to prevent the results from being ambiguous and ultimately unhelpful for the intended purpose. The protocol was registered and made publicly available on the Open Science Framework (OSF, https://osf.io/dzjpu, accessed on 1 July 2024).

### 2.2. Literature Sources and Research Strategy

Three authors (DB, SA, and JdK) independently performed the research process. In case of disputes or discrepancies between researchers, a senior author (ST) was consulted to give the final judgement and provide consensus. The search was performed between 1 March 2024 and 31 March 2024. The entire literature search strategy process is presented in detail as follows.

The research was conducted on MEDLINE, Scopus, and Web of Science. Keywords were selected using medical subject headings (MeSH) for PubMed and MeSH/EMTREE for Scopus. The keywords “TEVAR”, “thoracic aortic repair”, and “aortic coverage” were combined with item-specific secondary keywords to obtain the first publication cluster. When possible, the [MeSH terms] modality was used during query composition to avoid redundant results. The Boolean operators “AND” and “OR” were used to connect keywords with each other. Furthermore, a thorough examination of the reference lists of the selected studies was conducted to identify any additional relevant publications. Additionally, the “Article in press” sections of vascular journals to identify articles that had not yet been indexed in scientific databases was checked (see Appendix B). All original articles published in English and between 1 March 2004 and 1 March 2024 were included, specifically or secondarily treating the problem of length of coverage and DLZ level during TEVAR for TBAD.

### 2.3. Objectives

Specific PICO [7] questions were used to design the current study before the search. In particular, an attempt was made to answer 5 dominions about length of coverage/DLZ in TEVAR for TBAD patients (Table 1):

## 3. Results

### 3.1. Why Cover?

TBAD is a critical vascular disorder characterized by a tear in the inner layer of the descending thoracic aorta. This tear creates two distinct lumens within the aortic wall: the true lumen, which maintains normal blood flow, and the false lumen, formed by the dissection. This disruption in the aortic wall can severely compromise blood flow, leading to the potential malperfusion of vital organs or, in extreme cases, aortic rupture.

With a 5-year mortality rate ranging from 30% to 40% and an in-hospital mortality of around 14% [8,9,10], TBAD poses a significant threat to patient health. Early detection and appropriate management are essential for improving patient outcomes. Treatment strategies often involve a combination of medical therapies aimed at controlling blood pressure and heart rate along with surgical interventions when necessary. In contrast to Stanford type A aortic dissection (TAAD), however, the need for the surgical treatment of TBAD is affected by clinical presentation and radiological features [11].

If a patient is diagnosed with TBAD without any high-risk features or complications, treatment with the best medical treatment is advised. This treatment is aimed at lowering blood pressure and heartrate, and analgesic treatment is optimal [11].

Numerous risk factors have been identified that can exacerbate the progression of dissection, leading to subacute and delayed complications. Morphological risk factors are a primary entry tear in proximity of the left subclavian artery (LSA) or inner curvature [12,13,14], an entry tear >10 mm [13,15], a high antegrade flow volume in the false lumen [13], an aortic diameter of >40 mm [13,16], or a false lumen diameter >22 mm [16,17]. Clinical high-risk criteria are persistent pain and uncontrollable hypertension [18].

In the case of complicated TBAD (with signs of rupture and/or malperfusion) or TBAD with high-risk features, patients with a suitable anatomy are candidates for immediate TEVAR [11].

TEVAR is used to cover the entry tear and thereby exclude antegrade blood flow in the false lumen. By excluding the false lumen from further flow, the rupture of the false lumen can be avoided. Additionally, it can also restore true lumen blood flow, restore malperfusion and, in the long run, improve aortic remodelling, thereby reducing the risk of further disease progression and late complications or reinterventions.

The main reason for TEVAR in TBAD is to depressurize the false lumen by excluding blood flow into the false lumen, either antegrade or retrograde, as much as possible [19]. Continual blood flow into this false lumen can create acute or late complications. One of the most serious complications is a rupture of the aorta. This can occur when the pressure in the false lumen keeps increasing because of continual antegrade blood flow into the false lumen. The wall of the false lumen has less strength and is less thick than the wall of the native aorta [20,21]. This combination of increasing pressure and a more fragile aortic wall can cause a rupture of the false lumen. In the case of a rupture, immediate surgical treatment is necessary to repair the aortic wall and exclude the false lumen from blood flow by closing the entry tear to keep the patient alive.

Another serious complication is the static malperfusion of the visceral organs or limbs. If the aorta is dissected in such a way that one of the arteries supplying blood to the visceral organs or limbs originates from the false lumen, there is a possibility that this artery may fail to supply adequate blood to the corresponding organ [21,22]. Another cause of static malperfusion is the dissected septum physically closing off one of the branch artery origins, blocking blood flow from this artery and end organ. A different type of malperfusion is dynamic malperfusion, in which the dissected septum is flexible and can cause intermittent malperfusion by obstructing the vessel only in certain phases of the cardiac cycle [23]. Malperfusion can be life threatening and is also a reason for immediate surgical intervention. There are several organs that can be the victim of malperfusion and ischemia, visceral ischemia, renal ischemia, limb ischemia, and spinal cord ischemia. The ncessity for intervention is dependent on the severity and radiological features [24,25].

Later complications of TBAD are an extension or recurrence of the dissection, aneurysmal degeneration, and malperfusion. These patients are initially managed medically; in this group, it is uncommon to see the spontaneous healing of the dissection, classified as the complete thrombosis of the false lumen. Continual flow into the false lumen, either without thrombosis or partial thrombosis, has been shown to lead to a worse prognosis. The INSTEAD trial, while showing no statistically significant difference in overall survival at 2 years of follow-up, showed favourable aortic remodelling in 91.3% of the endovascularly treated group, as compared to the 19.4% of the medically treated patients [19]. A patent false lumen can lead to aneurysmal degeneration over time, which in some cases necessitates surgical repair to avoid complications. A patent false lumen can also lead to further dissection, which can be antegrade (in the direction of flow) or retrograde (against the direction of flow). A retrograde dissection might continue all the way into the aortic arch and/or ascending aorta, which might require major open surgery. The further extension of the dissection can also cause new malperfusion, in which case surgical repair is also indicated. The INSTEAD-XL trial, which included only stable type B aortic dissections after the best medical treatment, showed a favourable outcome of endovascular treatment versus optimal medical treatment in both aorta-specific mortality (6.9% vs. 19.3%) and the progression of the disease (27% vs. 46.1%) [26]. The ADSORB trial, which included only complicated type B dissections, compared the best medical treatment versus the best medical plus endovascular treatment and also reported significantly less events of the endpoint of incomplete/no false lumen thrombosis, aortic dilatation, or aortic rupture for those treated with TEVAR [27].

TEVAR itself has a high rate of procedural success, reported as high as 98%, with a decrease in 30-day mortality compared to open treatment from 29.3% to 2.8% [22]. This does not mean, however, that TEVAR is without risk. Three meta-analyses that reported on short- and mid-term results on TEVAR for complicated TBAD reported a stroke rate between 1.9% and 6.3% [28,29,30]. A multicentre European registry, the VIRTUE Registry, showed an 8% risk of stroke and 2% risk of spinal cord ischemia [31].

TEVAR itself can also cause long-term problems, such a distal stent graft-induced new entry (dSINE), where the distal end of the stent perforates the aortic flap and creates a new entry tear, which can allow for blood flow into the false lumen. The occurrence of dSINE has been reported in a systematic review to be 10.1% of all cases of TBAD treated with TEVAR [26]. The incidence of dSINE seemed to be lower in patients managed in the acute phase of TBAD as opposed to in the chronic phase because of the mobility of the dissected flap. Distal oversizing was also shown to be associated with dSINE [32,33].

Another problem in TEVAR procedures is the occurrence of endoleaks, in which blood still manages to enter the false lumen despite a stent being in situ. Endoleaks are classified based on the location of the leak. The occurrence of an intraoperative endoleak in TBAD treated with TEVAR is around 10% in complicated TBAD and around 1% in uncomplicated TBAD [34].

Migration is another possible problem of TEVAR for TBAD, where the stent is not attached to the aortic wall strongly enough and thereby moving under the forces of the blood flow. This could cause the entry tear to no longer be covered and cause further complications. One study showed a migration rate in all diseases treated with TEVAR of 7.3%, with a migration rate of 2.4% in TBAD specifically [34].

Hence, the treatment of TBAD with TEVAR is necessary to cover the proximal entry tear and to reduce the chance of acute and late complications.

### 3.2. How to Cover

Open surgical repair for TBAD is now largely reserved for patients with connective tissue disorders, such as Marfan syndrome or Ehlers-Danlos syndrome or when the patient’s anatomical characteristics make them unsuitable for endovascular procedures. Open repair is burdened by a higher risk of perioperative morbidity and mortality compared to TEVAR. Studies have consistently shown lower in-hospital mortality rates and improved short-term survival with TEVAR, positioning it as the safer choice for most patients presenting with complicated TBADs [19].

Preoperative imaging, primarily through computed tomography angiography (CTA), is essential to differentiate between the true and false lumen, identify primary and secondary entry tears, assess the aortic anatomy, identify high-risk features, and measure the extent of the dissection. The appropriate endograft is selected and is typically oversized by 0% to a maximum of 10% relative to the aortic diameter at the proximal landing zone to secure adequate radial force and prevent migration while reducing the risk of retrograde type A dissection. Additionally, the use of endografts with proximal bare metal stents is typically avoided when treating TBAD, as they are associated with a higher incidence of RTAD [35,36]. Excessive oversizing is associated with retrograde type A dissection (RTAD) when a proximal new entry tear is induced by the endograft. However, new entry tears can also occur distally and are defined as distal stent-induced new entry (dSINE) tears. RTAD occurs in about 2.3% of patients undergoing TEVAR for TBAD, with most cases emerging within the first 30 days post-procedure [37]. Significantly, the incidence is higher in patients with acute TBAD compared to those with chronic TBAD. Distal new entry tears can cause flow towards the false lumen, causing it to be pressurized, preventing its complete thrombosis. The incidence of dSINE varies greatly, ranging from 3.4% to as high as 27%, with higher rates often observed in patients treated for chronic aortic dissection [38]. These tears typically develop 12 to 36 months post-TEVAR, are usually asymptomatic, and are discovered during postoperative surveillance imaging [39].

The primary objective when deploying a TEVAR for TBAD is to cover the primary entry tear to reduce false lumen perfusion, promote its thrombosis, and re-perfuse aortic branch vessels in the case of malperfusion syndrome. The proximal landing zone of TEVAR should always coincide with a portion of the aorta unaffected by the dissection. In many cases, the coverage of the LSA is necessary to obtain an appropriate landing zone and should be accompanied by LSA revascularization in all non-emergent cases. When performing TEVAR for complicated TBAD with LSA coverage, immediate LSA revascularization can be deferred. Instead, careful monitoring for neurological deficits should be prioritized, and if any deficits are detected, LSA revascularization can then be performed as a secondary intervention.

During the procedure, identifying and accessing the true lumen is crucial, and the use of intravascular ultrasound (IVUS) is strongly recommended both to confirm true lumen access and for the following intraoperative evaluation of aortic branches and secondary entry tears [40].

Femoral access is most commonly used, but in cases of narrow iliac or femoral arteries, excessive tortuosity, or heavy calcification, alternative access routes or the use of a prosthetic conduit might be necessary.

The coverage of the aorta should extend to a point that adequately seals the primary entry tear and any significant secondary tears, ensuring the comprehensive management of the dissection. However, the challenge in determining the extent of coverage lies in balancing adequate dissection management with the risk of inducing complications, such as spinal cord ischemia, which can result from the extensive coverage of critical intercostal arteries [41]. The presence of intercostal arteries and the risk of spinal cord ischemia necessitate a meticulous and judicious assessment of the patient’s aortic anatomy. In cases requiring extensive coverage of the aorta, exceeding 150–200 mm strategies, such as cerebrospinal fluid drainage, are considered to mitigate the risks of paraplegia [4,5]

#### 3.2.1. PETTICOAT

The distal BMS expands the true lumen and reapproaches the separated aortic layers while maintaining essential blood flow to visceral arteries. This dual approach offers an enhanced management of both the true and false lumens and aims at stabilizing extensive dissections more comprehensively than TEVAR alone, favouring positive aortic remodelling [42].

The PETTICOAT technique showed beneficial effects in aortic remodelling, particularly in the re-expansion of the true lumen of the distal thoraco-abdominal aorta. This technique can be used as an additional tool to promote end organ perfusion in complicated TBADs [43]. A controlled study involving patients with Debakey type I and IIIb aortic dissections treated with a combined strategy of TEVAR and a thoracoabdominal BMS has highlighted the potential of this approach to promote aortic remodelling [44].

#### 3.2.2. e-PETTICOAT

The e-PETTICOAT technique was developed to treat TBADs extending into the iliac arteries, which may contribute to retrograde false lumen perfusion and aneurysmal progression. This technique involves deploying TEVAR to close proximal entry tears and a distal BMS across the distal thoracic and abdominal aorta. The additional steps compared to the standard PETTICOAT technique include the ballooning of the TEVAR and BMS to re-expand the true lumen and the placing of two covered stents as kissing iliac stent grafts extending within the abdominal BMS. This configuration has the aim of reapproaching dissection layers and promoting positive remodelling through long aorto-iliac segments [45]. The e-PETTICOAT technique has been applied successfully in acute and rapidly evolving extensive TBAD cases. While initial outcomes appear favourable, long-term follow-up shows declining survival rates and an increasing need for reinterventions. This suggests that although the e-PETTICOAT can be effective initially, it may not significantly influence long-term outcomes in patients with chronic TBAD [46].

**Figure 1:**
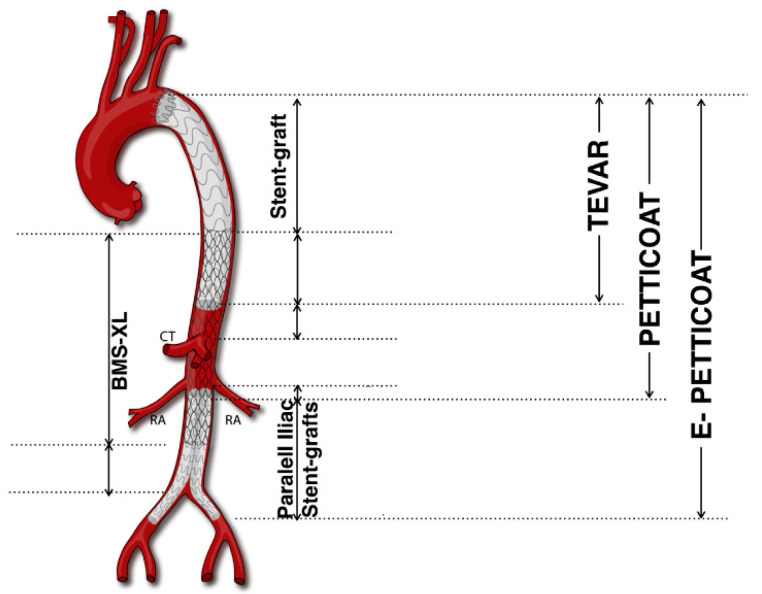
PETTICOAT and e-PETTICOAT technique. CT, Celiac Trunk; RA, Renal Artery; BMS, bare metal stent (Adapted from Kazimierczak et al. [45]).

The PETTICOAT technique (Provisional ExTension To Induce COmplete ATtachment), an extension of conventional TEVAR, specifically addresses extensive TBADs, notably those involving the visceral segment. First described in 2006, PETTICOAT employs a standard TEVAR device to seal the proximal entry tear, complemented by a distal bare metal stent (BMS) (Figure 1).

#### 3.2.3. STABILISE

The STABILISE (Stent-Assisted Balloon-Induced Intimal Disruption and Relamination) technique is an endovascular strategy developed as an additional tool in the treatment of acute and subacute TBAD. This technique builds upon the foundation laid by TEVAR and PETTICOAT, incorporating additional steps to address limitations in achieving complete false lumen obliteration. The procedure’s goal is to create a single-channelled aorta and to promote extensive aortic remodelling. Initially, a conventional stent graft is deployed to cover the proximal entry tear, directing blood flow back into the true lumen and initiating the thrombosis of the false lumen, as described for TEVAR. Following this, BMSs are placed distally, extending into the thoracoabdominal aorta. The critical step involves the use of a compliant balloon inside the stent graft to expand it to its nominal diameter followed by ballooning with non-compliant or semi-compliant balloons of the BMS to disrupt the dissection membrane, which encourages the expansion of the true lumen and aids in the relamination process, effectively turning a multi-luminal dissection into a unified aortic channel. While the STABILISE technique demonstrates a promising approach to managing acute and subacute TBADs by potentially improving long-term aortic remodelling and reducing reintervention rates, the available literature reflects a cautious optimism. Studies indicate a notable rate of complete false lumen obliteration and suggest better control over aortic diameter evolution compared to traditional TEVAR alone. However, the clinical success and safety of the technique require further validation through more extensive and longer-term studies to establish definitive conclusions about its effectiveness and safety profile [47,48,49].

#### 3.2.4. Knickerbocker

The Knickerbocker technique was first described in 2014 as a strategy developed to prevent aortic aneurysmal degeneration caused by persistent FL perfusion following TBADs [50]. In this procedure, TEVAR is performed covering the proximal entry tear as described previously with the additional deployment of an oversized stent graft in the DTA. The additional stent graft is balloon dilated in its mid-section, thus disrupting the dissection membrane, blocking retrograde FL flow from distal re-entry tears. The Knickerbocker technique is typically applied during the subacute phase of TBAD, when the distal DTA is not excessively dilated and has shown promising results in promoting positive aortic remodelling in the thoracic aorta [51].

#### 3.2.5. False Lumen Treatment—Candy Plug

The candy plug technique was first developed in 2013 for chronic TBAD management to induce false lumen thrombosis and positive aortic remodelling. It involved the modification of a thoracic stent graft with the addition of a diameter-reducing suture in the middle section of the graft. This modification creates a narrowed central section that can accommodate a 20 mm Amplatzer Vascular Plug II [52]. The candy plug is deployed into the false lumen, following the placement of a thoracic stent graft into the true lumen, extending down until just above the celiac trunk. This strategic placement is crucial to prevent perfusion of the false lumen of the thoracic aorta. The newest self-occluding candy plug III (CP III) is a custom-made thoracic stent graft featuring three independent nitinol Z-stents covered by woven polyester fabric. A distinctive feature of the device is its distal sleeve segment made of fabric, which tapers down in diameter. This design allows for the retraction of the dilator tip after deployment while preventing retrograde blood flow into the false lumen [53].

#### 3.2.6. False Lumen Treatment—Embolization

This method involves deploying embolic materials such as coils, plugs, and glue into the FL to obstruct blood flow and encourage its thrombosis. A variety of devices have been used to embolize the FL with preliminary positive results in terms of FL thrombosis and feasibility [54].

The “cork in the bottle neck” technique was first described by Loubert et al. in 2003, and it employed placing large occlusive devices along with detachable balloons and thrombin directly into the false lumen. This setup aims at blocking the retrograde flow into the FL, effectively sealing it [55]. This technique has largely been replaced by the candy plug, which is specifically designed to achieve the same outcome with greater efficiency and reliability. Various small case series have adopted similar approaches using coils, cyanoacrylate glue, and iliac occludes, demonstrating the technique’s adaptability and feasibility, although it is generally more suited to patients with smaller false lumen diameters at the level of the diaphragm due to the limitations of the available embolic materials for larger diameters [56].

### 3.3. Who to Cover

#### 3.3.1. Complicated and Uncomplicated Type B Aortic Dissection

Traditionally, uncomplicated TBAD has been treated with “anti-impulse therapy” with strict blood pressure control and follow-up CT-scans. However, more than three quarters of patients develop post-dissection aneurysms requiring surgical treatment [57]. Given its efficacy and low morbidity, TEVAR has been increasingly performed in uncomplicated patients, with favourable mid-term results by promoting FL remodelling and preventing late aneurysmal degeneration [26,27]. Despite promising outcomes after TEVAR in acute uncomplicated TBAD, it remains unclear if all uncomplicated patients should be treated. Reintervention rates remain substantial, with 26% of patients requiring reintervention for endoleaks, distal fenestrations, and metachronous pathology over a median follow-up of 34 months [58].

While there is consensus on treating the primary entry tear in the DTA, the optimal length of coverage remains unclear. Up to 200 mm coverage is generally considered safe, [4] but other factors, such as previous abdominal aortic surgery with lumbar arteries ligation, occluded the hypogastric artery, and LSA should be considered when evaluating the risk of SCI.

Given the lack of definitive clinical evidence, a patient-specific approach is advised for acute uncomplicated TBAD, considering anatomic suitability and high-risk factors for pathology progression (Table 2). Stratifying patients into low-, intermediate-, and high-risk categories can aid in decision-making regarding the treatment approach and extent [59].

TEVAR has become the gold standard in treating complicated acute TBAD, aiming to cover the proximal entry tear (or the ruptured aorta) and promote FL thrombosis to reverse end-organ ischemia. The success of endovascular management largely depends on factors such as the location of the primary entry tear and the longitudinal extent of the dissection. If branch vessel obstruction persists after TEVAR, percutaneous stenting is usually effective at restoring blood flow. Temporal changes in the plasticity and compliance of the dissection membrane significantly impact long-term success. Acute dissections remodel more rapidly and extensively, especially in more proximal aortic segments covered by the stent graft [59]. For dissections confined to zones 2–5, TEVAR with LSA revascularization is usually effective, while dissection involving zones 6–11 may necessitate subsequent interventions of distal re-entry tears (e.g., candy plug, coil embolization, or Knickerbocker technique) due to the enlargement of the untreated aortic segments.

In TEVAR for acute complicated TBAD, Manning et al. observed a higher rate of reintervention in “short coverage” patients (entry tear coverage) and began to routinely deploy the stent graft down to the diaphragm level [60]. Conversely, in patients with intramural hematoma (IMH), less extensive coverage was beneficial. Xue et al. investigated the association between aortic coverage extent and remodelling after TEVAR in 201 patients with acute (67%) and chronic (33%) TBAD. The mean stent graft length was 157 ± 33 mm. More extensive coverage was independently associated with a lower risk of thoracic aortic expansion and increased FL thrombosis [61]. Lastly, Lou et al. treated 91 acute TBAD patients, reporting similar results: extended coverage (up to the celiac artery) showed higher FL obliteration rates, with similar SCI risk [62]. All authors recommended SCI prevention strategies with extended treatment, especially LSA revascularization and cerebral fluid drainage, avoiding hypotension and hypoxia.

#### 3.3.2. Chronic Dissection with Aortic Dilatation/Aneurysm

After the initial diagnosis of TBAD, post-dissection aortic aneurysm (PDAA) may develop, often extending into the thoracoabdominal aorta. Indications for elective repair include large size (>60 mm), rapid enlargement (>10 mm/year), or compression symptoms. TEVAR can fail due to continued FL pressurization from uncovered distal fenestrations and the thickening of the chronic flap, which hampers remodelling. While it is an option to cover only a limited thoracic aorta segment and monitor the abdominal aorta with regular scans, the current standard is to cover most of the thoracic aorta. Qing et al. noted that short aortic coverage (<162 mm) might result in unfavourable aortic remodelling [63]. Iida et al. analysed residual dissection following type A aortic dissection repair and found that persistent tear in the DTA hindered remodelling, requiring additional TEVAR to prevent adverse aortic events. Interestingly, no secondary reintervention or open conversion was observed during the average 22-month follow-up [64]. Similarly, Verma and colleagues found comparable results in their analysis of 38 patients who underwent TEVAR for TBAD: longer aortic coverage (>200 mm) was associated with improved aortic remodelling without significantly increasing the risk of SCI [65]

While mid-term outcomes in properly selected patients show good results, long-term outcomes and risk factors for endovascular treatment failure await further elucidation. Branched and fenestrated EVAR (BEVAR and FEVAR) hold promising opportunities for addressing thoracoabdominal aorta issues, but they face technical challenges. Nonetheless, advancements in endovascular techniques for chronic PDAA might expand their applicability and improve long-term outcomes.

#### 3.3.3. Connective/Congenital Disease

Although feasible, the use of TEVAR in patients with connective tissue disorders (CTD) remains contraindicated [3,5]. The fragility of the aortic wall in CTD patients leads to the unavoidable and continuous dilation of the aortic diameter, with high rates of reinterventions and increased risks of stent graft-related complications with uncertain long-term outcomes [66,67]. Additionally, as CTD was an exclusion criterion for pivotal trials leading to all FDA-approved stent grafts, controversy remains regarding the benefits and risks of stent graft deployment in CTD patients. For these reasons, there is currently a consensus that TEVAR should be limited to exceptional cases and emergency situations in these patients [68], serving as a bridge to definitive surgery [69,70]. Malperfusion syndrome appears to be more common in CTD patients. While aortic fenestration via balloon septostomy might be considered to address malperfusion, TEVAR may be the only life-saving option in the case of a rupture, complex dissection anatomy, and multiple malperfused territories in emergent settings. Moreover, many CTD patients will require aortic branch stenting in addition to aortic repair or TEVAR to alleviate malperfusion upon presentation. To date, there is no consensus on the management of thoracoabdominal aortic aneurysm in chronic dissection or after the initial conventional surgical repairs in CTD patients. However, future studies might reveal significant benefits and insights from BEVAR and FEVAR for this group of patients. Several technical considerations should be considered to prevent stent graft-related complications, such as retrograde aortic dissection and SINE. IVUS may provide valuable information for device delivery, careful guidewire placement, and sizing. It is crucial to avoid excessive oversizing and the use of devices with proximal bare springs. Studies on stent graft-induced tears are generally based on small study populations, making it challenging to determine the optimal length of stent grafting at the index operation and the choice of the distal landing zone. In many CTD patients, the aorta will be relatively straight, suggesting that a more distal landing zone in the mid-to-distal third may reduce conformability issues. However, the use of cerebrospinal fluid drainage should be considered to mitigate the risk of SCI.

### 3.4. Computational Studies

**Figure 2:**
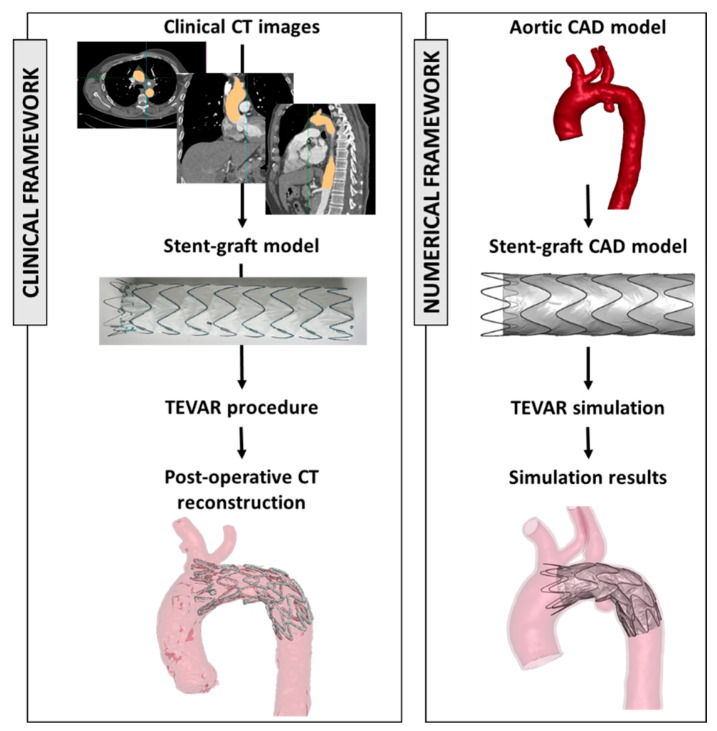
On the left, clinical workflow from CT images and the selection of the appropriate device size and the postoperative evaluation. On the right, the reconstruction of the aortic model (from CT images) and stent graft model and the results of the numerical simulation. The figure is adapted from 2023, Ramella et al. [72].

In the recent literature, numerical models have been demonstrated to be a valuable tool for investigating clinical procedures. These models allow researchers to simulate various scenarios, aiding in the understanding of complex interactions between the aortic district and the implanted device [71,72,73]. Figure 2 shows the framework to extract engineering features starting from clinical images and device models.

In the context of TEVAR modelling for the treatment of aortic dissections, some studies have been identified that address the effect of different stent graft lengths on the procedural outcome (Table 3).

First, in 2018, Ma et al. [74] published a study investigating the effect of the increased stent graft length and oversizing on the stress exerted on the aortic wall within a patient-specific acute TBAD model. They replicate the TEVAR procedure using two commercial stent graft models, combining different diameters and lengths with a total of six cases. A major stress increase was found in the aortic wall due to the higher device oversizing. No statistical evidence of change in stress distribution was encountered by increasing the device covering length. Similar results were discussed in 2021 by Tan et al. [75], which carried out numerical simulations on six patient-specific aortic dissection models, of which three developed dSINE and three did not. Their study aimed to explore the interplay between anatomical characteristics, biomechanical variables, and their potential relationship with dSINE formation. Aortic anatomies were reconstructed from postoperative CTA images, and the device was assumed to be fully conformed to the aorta wall surface (i.e., no device simulated but material properties of the aortic wall are changed where the stent graft is supposed to be). Anatomical factors and implanted devices varied among each patient. Pressurization simulations were carried out to assess the stress distribution in the aortic wall. Across all the patients, maximum stress regions were identified in the distal region of the aorta, where dSINE had developed, but they were mainly related to device oversizing. Additionally, idealized anatomies were also considered to examine the impact of SG length, revealing no correlation between simulated lengths and changes in stress distribution.

Instead, in 2021, Kan et al. [76] proposed a study on the analysis of the impact of varying stent graft lengths on the stress distribution generated on the intimal dissection flap and aortic wall of a patient-specific TBAD model. They modelled a commercial stent graft with three different covering lengths (short, medium, and long). In the clinical scenario, the patient was treated with the shorter device. As a result, when simulating the shorter stent, they observed a spatial correlation of high-stress regions in the DLZ, coinciding with the development of dSINE during follow-up. Also, high stresses were registered in the PLZ, where a new tear occurred as well. Conversely, simulations with increased stent lengths revealed a notable reduction in wall stress within the DLZ (up to 60% compared to the short case), suggesting that longer stent grafts may mitigate the development of dSINE. The same group in 2024 [77] proposed a similar analysis on the identical patient-specific TBAD model considering this time both three different SG lengths and different device designs, with the final goal of evaluating the biomechanical implications of three commonly used stent designs for TEVAR in terms of morphological changes and stress distribution. From a morphological point of view, the analysis revealed that the design influenced the cross-sectional enlargement of the true lumen. Regarding the stress generation on the intimal flap, short stent grafts generated higher stresses, located mostly in the DLZ, confirming the results of the previously mentioned studies. It is essential to note that all of the cited studies focused either on a single patient or on a small cohort of patients. Expanding the patient cohort could improve the applicability of the results and offer statistical evidence of the findings.

## 4. Discussion

Upon conducting this comprehensive scoping review, it becomes evident that although numerous manuscripts have been published on the subject of TEVAR with regard to various techniques, complications, tips, and outcomes, the availability of specific data pertaining to the optimal length of coverage during endovascular repair for TBAD, particularly in the context of the DLZ, remains remarkably scarce. The DLZ, being a crucial aspect in the planning of TEVAR, assumes significant importance due to its association with several postoperative complications, such as dSINE, distal dissection, and SCI. Moreover, stent coverage to the level of the diaphragm and the level of celiac artery have been considered independent predictors of the distal aortic segmental enlargement, with a consequent increase in the postoperative long-term mortality rate [78]. Furthermore, this specific landing zone can also contribute to graft displacement, especially in cases where the native shape of the aorta exhibits tortuosity [79].

It is indisputable that numerous studies have put forth qualitative analyses, delving into the examination of the relationship between TEVAR coverage and the occurrence of postoperative complications. Nonetheless, a definitive quantitative estimation regarding the optimal length of the endoprosthesis has yet to be substantiated. Some specific and useful data have been provided by the above-mentioned studies by Xue and collaborators [51], in which the percentage of stented descending aorta (PSDA) was used to measure the extent of stent graft coverage, dividing patients into two groups based on the median PSDA: a lower group (≤31.3%) and a higher group (>31.3%). Otherwise, Lou et al. classified their cohort as standard or extended (endograft coverage of the entire DTA extending below the diaphragm to the level of the celiac artery) TEVAR [62].

Conversely, there being so many variables to take into consideration during the planning of the procedure, it remains difficult to have a timely and precise estimate of the “right” length of the endoprosthesis. Indeed, several characteristics should be considered: some patient-related (inguinal access-, sex-, comorbidity-, and other baseline non-aorta-related characteristics), some aorta-related (landing zone-, spinal artery-, and TBAD feature-related) and some endograft-related (type-, stiffness-, conformability-related…).

Moreover, also in the postoperative period, several factors can influence long-term outcomes, such as drag forces, postoperative patient’s comorbidities, and interventions.

This review has shed light on the intricate nature of planning TEVAR for TBAD, aiming to thoroughly examine the key components involved in the decision-making process preceding the TBAD repair using endovascular techniques. All these features are evaluated to provide the best balance of risks/benefits linked to the intervention. The length of coverage could be one of the main keys to better evaluate the effectiveness of TEVAR in TBAD.

Despite these limitations, reporting the percentage of covered aorta could normalize differences among patients, providing useful insights for future research. This measurement could be defined as the percentage of DTA covered by the endograft from LSA to celiac trunk, using a centerline analysis from preoperative CTA.

Recently, the utilization of numerical models has been demonstrating their value as an effective tool for investigating various technical aspects in endovascular techniques. These models serve as a means for researchers to simulate different scenarios, which in turn aids in the comprehension of the complex interactions that occur between the aortic district and the implanted device. While the integration of simulations into the preoperative decision-making process for every TEVAR procedure may still be a distant prospect (despite not so distant studies! [80]), it is crucial to acknowledge the growing importance of these analyses. Their significance cannot be overstated, as they have the potential to revolutionize the way clinicians’ approach and plan for TEVAR interventions.

## 5. Conclusions

The determination of the appropriate length of coverage in TEVAR for TBAD patients is influenced by various factors, including the patient’s initial characteristics, specific TBAD-related features, the type of endoprosthesis employed, and the behaviours of the graft post-procedure. However, it is important to acknowledge that there is a dearth of robust data available to establish the optimal length of TEVAR for TBAD definitively. Despite this limitation, it remains crucial to underscore the significance of reporting the percentage of the aorta that is covered during the procedure. Moreover, computational studies hold promise in enhancing our understanding and improving postoperative outcomes. By further advancing and exploring computational modelling, we can strive to optimize the length of coverage and ultimately enhance patient outcomes in the context of TEVAR for TBAD.

## Figures and Tables

**Table 1:** Summary of the main PICO questions.

Why cover? How long should my aortic coverage be to exclude TBAD and minimize its progression?
Who to cover? Which patients and/or clinical conditions can derive the greatest advantages from specific aortic coverage in TEVAR for TBAD patients?
How to cover? How can the consideration of aortic coverage analysis be incorporated into the operative technique choice to enhance outcomes following TEVAR for TBAD?
How much to cover?Is there specific evidence from the literature on the length of aortic coverage in TEVAR for TBAD patients?
Future perspectives Can ex-vivo and artificial/computational analysis predict the optimal length of coverage in TEVAR for TBAD patients?

**Table 2:** Type B aortic dissection patients’ risk factors.

Uncomplicated	Complicated	High-Risk
No high-risk features	Malperfusion	False lumen diameter > 22 mm
No malperfusion	Rupture	Aortic diameter > 40 mm
No rupture		Radiographic malperfusion
		Refractory pain
		Bloody pleural effusion
		Refractory hypertension
		Primary entry tear on the inner curve
		Readmission

**Table 3:** Reporting main results from computational studies regarding graft length and characteristics in type B aortic dissection patients. TBAD, type B aortic dissection patients; SG, stent graft; SINE, stent induced new entry tear; CTA, computed tomography angiography; FEA, Finite Element Analysis.

Manuscript	Aim of the Study	Aortic Model	Stent Graft Models	Main Findings
2018, Ma et al. [74]	Evaluate the stress distribution of the aorta changing SG configurations	Patient-specific acute TBAD	Two stent graft designs with different diameters and lengths. Diameters: 33 mm, 38 mm Length: 130 mm, 140 mm, 172.5 mm.	Major effect due to increased oversing. No correlaton found by changing the length of the stent graft. Reducing length leads to slight decrease in the stress, but no statistical evidence.
2021, Tan et al. [75]	Study von Mises stresses and shear elastic strain after TEVAR in relation to the development of distal SINE.	Six patient-specific TABD anatomies, each one with a different stent graft. And 6 idealised anatomies.	Patient-specific: different stent graft lengths, based on the postoperative CTA. Ideal: one single anatomy with different stent graft lengths.	Patient-specific: High stress regions are identified in the distal region of the aorta, where distal SINE has developed. Idealised: no correlation on the stress distribution changing the length. However, stresses increased with the increase of tortuosity.
2021, Kan et al.[76]	Analysis of the effect of different stent graft length on the stress distribution on the intimal dissection flap and aortic wall. Correlation with SINE location.	Patient-specific anatomy with TBAD.	Models of the Zenith TX2 PT device. Length studied: 158 mm (short), 208 mm (long), 183 mm (medium)	Short stent: spatial correlation of the high stress regions in the distal LZ, where the distal SINE was found in the follow-up, and proximal LZ where new tear occurs in the follow-up. Increasing length lead to a better sealing of tear, reduction of wall stress in the distal landing zone (up to 60% compared to the short case). Longer SG may reduce the development of SINE.
2024, Kan et al.[77]	Evaluate the biomechanical effects induced on the vessel by three different stent designs commonly used for TEVAR in terms of aorta morphological changes and stress distribution.	Pateint specific TBAD.	Three stent graft models with different designs and materials. Each of them in a short (15 cm) and long (20 cm) configurations.	Morphological analysis. all the stents: enlargement on the true lumen. The desing and materilas matter: SG1 induced the most abrupt increase in the cross-section area, followed by SG2 and SG3. Stress analysis. The design and materials matter: SG1 generates higher stresses on the intimal flap (followed by SG2 and SG3). Short geometries generates hogher stresses as in the study above.

## Data Availability

No new data were created/presented.

## Appendix A

Preferred Reporting Items for Systematic Reviews and Meta-Analyses extension for Scoping Reviews (PRISMA-ScR) Checklist.

**Section**	**Item**	**Prisma-ScR Checklist Item**	**Reported on Page #**
**Title**			
Title	1	Identify the report as a scoping review.	1
**Abstract**			
Structured summary	2	Provide a structured summary that includes (as applicable): background, objectives, eligibility criteria, sources of evidence, charting methods, results, and conclusions that relate to the review questions and objectives.	3
**Introduction**			
Rationale	3	Describe the rationale for the review in the context of what is already known. Explain why the review questions/objectives lend themselves to a scoping review approach.	4, 5
Objectives	4	Provide an explicit statement of the questions and objectives being addressed with reference to their key elements (e.g., population or participants, concepts, and context) or other relevant key elements used to conceptualize the review questions and/or objectives.	5
**Methods**			
Protocol and registration	5	Indicate whether a review protocol exists; state if and where it can be accessed (e.g., a Web address); and if available, provide registration information, including the registration number.	6
Eligibility criteria	6	Specify characteristics of the sources of evidence used as eligibility criteria (e.g., years considered, language, and publication status), and provide a rationale.	6
Information sources *	7	Describe all information sources in the search (e.g., databases with dates of coverage and contact with authors to identify additional sources), as well as the date the most recent search was executed.	6
Search	8	Present the full electronic search strategy for at least 1 database, including any limits used, such that it could be repeated.	6
Selection of sources of evidence ^†^	9	State the process for selecting sources of evidence (i.e., screening and eligibility) included in the scoping review.	6
Data charting process ^‡^	10	Describe the methods of charting data from the included sources of evidence (e.g., calibrated forms or forms that have been tested by the team before their use, and whether data charting was done independently or in duplicate) and any processes for obtaining and confirming data from investigators.	6
Data items	11	List and define all variables for which data were sought and any assumptions and simplifications made.	6
Critical appraisal of individual sources of evidence ^§^	12	If done, provide a rationale for conducting a critical appraisal of included sources of evidence; describe the methods used and how this information was used in any data synthesis (if appropriate).	Not applicable
Synthesis of results	13	Describe the methods of handling and summarizing the data that were charted.	6
**Results**			
Selection of sources of evidence	14	Give numbers of sources of evidence screened, assessed for eligibility, and included in the review, with reasons for exclusions at each stage, ideally using a flow diagram.	7–19
Characteristics of sources of evidence	15	For each source of evidence, present characteristics for which data were charted and provide the citations.	7–19
Critical appraisal within sources of evidence	16	If done, present data on critical appraisal of included sources of evidence (see item 12).	7–19
Results of individual sources of evidence	17	For each included source of evidence, present the relevant data that were charted that relate to the review questions and objectives.	7–19
Synthesis of results	18	Summarize and/or present the charting results as they relate to the review questions and objectives.	7–19
**Discussion**			
Summary of evidence	19	Summarize the main results (including an overview of concepts, themes, and types of evidence available), link to the review questions and objectives, and consider the relevance to key groups.	19–21
Limitations	20	Discuss the limitations of the scoping review process.	21
Conclusions	21	Provide a general interpretation of the results with respect to the review questions and objectives, as well as potential implications and/or next steps.	19–21
**Funding**			
Funding	22	Describe sources of funding for the included sources of evidence, as well as sources of funding for the scoping review. Describe the role of the funders of the scoping review.	21
	JBI = Joanna Briggs Institute; PRISMA-ScR = Preferred Reporting Items for Systematic reviews and Meta-Analyses extension for Scoping Reviews. * Where sources of evidence (see second footnote) are compiled from, such as bibliographic databases, social media platforms, and Web sites. ^†^ A more inclusive/heterogeneous term used to account for the different types of evidence or data sources (e.g., quantitative and/or qualitative research, expert opinion, and policy documents) that may be eligible in a scoping review as opposed to only studies. This is not to be confused with information sources (see first footnote). ^‡^ The frameworks by Arksey and O’Malley (6) and Levac and colleagues (7) and the JBI guidance (4, 5) refer to the process of data extraction in a scoping review as data charting. ^§^ The process of systematically examining research evidence to assess its validity, results, and relevance before using it to inform a decision. This term is used for items 12 and 19 instead of “risk of bias” (which is more applicable to systematic reviews of interventions) to include and acknowledge the various sources of evidence that may be used in a scoping review (e.g., quantitative and/or qualitative research, expert opinion, and policy document). From: Tricco A.C., Lillie E., Zarin W., O’Brien K.K., Colquhoun H., Levac D., et al. PRISMA Extension for Scoping Reviews (PRISMAScR): Checklist and Explanation. Ann Intern Med. 2018;169:467–473. doi: 10.7326/M18-0850.		

## Appendix B

Journals screened to detect article in press.

European Journal of Vascular and Endovascular Surgery; Journal of Vascular Surgery; Annals of Vascular Surgery, Angiology, Circulation; The Journal of Cardiovascular Surgery; International Angiology; The Journal of Thoracic and Cardiovascular Surgery; JCTVS Open; JTCVS Techniques; Journal of Vascular Surgery Cases, Innovations, and Techniques.
